# Trends in US Hospital Admissions for Skin and Soft Tissue Infections

**DOI:** 10.3201/eid1509.081228

**Published:** 2009-09

**Authors:** John Edelsberg, Charu Taneja, Marcus Zervos, Nadia Haque, Carol Moore, Katherine Reyes, James Spalding, Jenny Jiang, Gerry Oster

**Affiliations:** Policy Analysis Inc., Brookline, Massachusetts, USA (J. Edelsberg, C. Taneja, J. Jiang, G. Oster); Henry Ford Health System, Detroit, Michigan, USA (M. Zervos, N. Haque, C. Moore, K. Reyes); Astellas Pharma US, Inc., Deerfield, Illinois, USA (J. Spalding)

**Keywords:** Skin infections, soft tissue infections, hospital admissions, staphylococci, methicillin-resistant Staphylococcus aureus, MRSA, bacteria, antimicrobial resistance, United States, dispatch

## Abstract

Using data from the 2000–2004 US Healthcare Cost and Utilization Project National Inpatient Sample, we found that total hospital admissions for skin and soft tissue infections increased by 29% during 2000–2004; admissions for pneumonia were largely unchanged. These results are consistent with recent reported increases in community-associated methicillin-resistant *Staphylococcus aureus* infections.

During 1998–2004, *Staphylococcus aureus* was the most common cause of skin and soft tissue infections (SSTIs) in North America; frequency of these infections was 44.6%, and the rate of methicillin resistance among the isolates was 35.9% (*1*). Over the past decade, community-associated methicillin-resistant *S. aureus* (CA-MRSA) has become a notable public health problem; it accounts for 14% of invasive infections nationwide and 59% (range 15%–74%) of SSTIs among patients seeking treatment at emergency departments in 11 US cities (*2*,*3*). The emergence of CA-MRSA infections may have resulted in increased numbers of hospitalizations for SSTIs because of an increasing incidence of antimicrobial drug failure in outpatient treatment and more aggressive approaches to the management of these infections by physicians who are aware of the heightened risk of becoming infected with CA-MRSA. To determine whether hospital admissions for CA-MRSA are increasing, we analyzed data from the Healthcare Cost and Utilization Project National Inpatient Sample.

## The Study

This study was based on data from the Healthcare Cost and Utilization Project National Inpatient Sample (HCUP NIS) for the 5-year period 2000–2004. HCUP NIS is a stratified random sample from ≈20% of all US community hospitals. For 2004, the NIS contains all discharge data from >1,000 acute-care hospitals in 37 states, representing >8 million hospital stays.

We identified all hospital admissions in the HCUP NIS for which a principal diagnosis of SSTI was given. As defined by the Uniform Hospital Discharge Data Set, principal diagnosis is the condition “established after study to be chiefly responsible for occasioning the admission of the patient to the hospital for care” (http://wonder.cdc.gov).

SSTIs were defined as the following: 1) acute lymphadenitis (International Classification of Diseases, 9th Revision, Clinical Modification [ICD-9-CM] 683.XX); 2) carbuncle and furuncle (680.XX); 3) cellulitis and abscess of finger and toe (681.XX); 4) impetigo (684.XX); 5) infection (chronic) of amputation stump (997.62); 6) other cellulitis and abscess (682.XX); 7) other local infections of skin and subcutaneous tissue (686.XX); 8) abscess of anal and rectal regions (566); 9) chronic ulcer of other specified sites (707.8); 10) chronic ulcer of unspecified site (707.9); 11) decubitus ulcer (707); 12) infection due to other vascular device implant and graft (996.62); 13) pilonidal cyst with abscess (685); 14) postoperative wound infection (998.5X); 15) posttraumatic wound infection, not elsewhere classified (958.3); 16) ulcers of lower limbs, except decubitus (707.1X); 17) gangrene (785.4); and 18) necrotizing fasciitis (728.86).

To aid in interpretation of the data, we grouped the above-listed SSTIs into 3 mutually exclusive categories: 1) superficial infections predominantly caused by *S. aureus* or *Streptococcus pyogenes* (groups 1–7 above); 2); deeper or healthcare-associated infections more likely to involve anaerobic or gram-negative organisms (groups 8–16); and 3) infections typically associated with a high rate of mortality (groups 17–18). For each year of interest, all SSTI admissions were then stratified by type of infection and selected patient and hospital characteristics.

To provide a benchmark against which to interpret possible trends in SSTI-related hospital admissions, we generated similar series for all admissions with a principal diagnosis of infectious pneumonia, another common type of infection that frequently results in hospitalization but for which no trends were expected. Infectious pneumonia was defined to include 1) pneumococcal pneumonia (ICD-9-CM 481); 2) other bacterial pneumonia (482.XX) 3); pneumonia caused by other specified organism (483.X) 4); bronchopneumonia, organism unspecified (485); and 5) pneumonia, organism unspecified (486).

Analyses were directed at discerning possible trends in SSTI-related hospital admissions at US acute-care hospitals over the 5-year period of study. Total SSTI admissions were estimated for each year by type of infection (i.e., superficial, deeper or healthcare-associated, often-fatal). Results also were stratified according to patient (e.g., age, sex) and hospital characteristics (e.g., urban vs. rural) available in the HCUP NIS. For comparison, we also examined total hospital admissions for infectious pneumonia.

Because the HCUP NIS undergoes periodic changes in its sampling frame and weighting method, as well as in data elements and definitions, we used the NIS Trends Supplemental (NIS-Trends) files to ensure comparability of data across the years of interest. The NIS-Trends files contain sampling weights and data elements that are consistently defined across all years of interest.

Analyses were primarily descriptive (i.e., no a priori hypotheses were made, and our results were not subjected to formal significance testing). All analyses were conducted by using the SAS Proprietary Software, Release 9.1 (SAS Institute, Cary, NC, USA).

The estimated total number of annual SSTI admissions to US acute-care hospitals rose steadily over the 5-year period, from 675,000 in 2000 to 869,800 in 2004, an increase of 194,000 (29%) admissions. In contrast, total admissions for infectious pneumonia fluctuated from year to year and were largely unchanged over this period (Table 1).

**Table 1:** Estimated total number of US hospital admissions for SSTIs and infectious pneumonia, 2000–2004*

Principal diagnosis	2000	2001	2002	2003	2004	Change from 2000 to 2004
SSTI	674,939	701,672	757,858	810,768	869,777	194,838 (+28.9%)
Infectious pneumonia	1,202,387	1,177,972	1,229,204	1,272,686	1,172,304	–30,083 (–2.5%)

*SSTI, skin and soft tissue infection. Source: Healthcare Cost and Utilization Project National Inpatient Sample, 2000–2004.

The increase in SSTI admissions was greatest among younger (age <65 years) rather than older patients (age 65–100 years) (37% vs. 14%, respectively) and for urban rather than rural hospitals (32% vs. 11%) (Table 2). The increase in SSTI admissions also was greatest among patients with superficial infections (e.g., cellulitis, abscess) rather than deeper or healthcare-associated infections (e.g., postoperative wound infection, infection due to vascular device) (33% vs. 24%).

**Table 2:** Number of hospital admissions with principal diagnosis of SSTI, by patient and infection characteristics and year*

Characteristics	Year				
	2000 (N = 674,939)	2001 (N = 701,672)	2002 (N = 757,858)	2003 (N = 801,768)	2004 (N = 869,777)
Age, y†					
<15	38,420 (5.7)	38,741 (5.5)	44,559 (5.9)	49,902 (6.2)	56,909 (6.5)
15–44	176,574 (26.2)	175,921 (25.1)	197,142 (26.0)	214,606 (26.5)	240,971 (27.7)
45–64	212,367 (31.5)	219,222 (31.2)	247,079 (32.6)	267,556 (33.0)	289,715 (33.3)
>65	247,544 (36.7)	267,768 (38.2)	269,048 (35.5)	277,828 (34.3)	281,288 (32.3)
Sex†					
Male	341,040 (50.5)	347,784 (49.6)	381,763 (50.4)	406,525 (50.1)	443,325 (51.0)
Female	333,771 (49.5)	353,849 (50.4)	376,075 (49.6)	400,737 (49.4)	423,255 (48.7)
Race†					
White	364,634 (54.0)	368,901 (52.6)	372,013 (49.1)	409,037 (50.5)	439,295 (50.5)
Black	81,654 (12.1)	81,299 (11.6)	94,307 (12.4)	99,892 (12.3)	111,416 (12.8)
Hispanic/Latino	57,217 (8.5)	59,345 (8.5)	63,657 (8.4)	78,091 (9.6)	75,142 (8.6)
Asian or Pacific Islander	6,457 (1.0)	7,219 (1.0)	8,496 (1.1)	9,366 (1.2)	10,053 (1.2)
Native American	2,082 (0.3)	2,375 (0.3)	2,211 (0.3)	1,595 (0.2)	3,890 (0.5)
Other	11,873 (1.8)	11,233 (1.6)	16,304 (2.2)	14,135 (1.7)	13,937 (1.6)
Infection type (ICD-9-CM diagnosis code[s])					
Superficial infections	390,158 (57.8)	404,536 (57.7)	440,501 (58.1)	479,222 (59.1)	520,099 (59.8)
Acute lymphadenitis (683.XX)	4,570 (0.7)	4,341 (0.6)	4,230 (0.6)	4,324 (0.5)	4,291 (0.5)
Carbuncle and furuncle (680.XX)	1,001 (0.2)	1,182 (0.2)	1,349 (0.2)	1,900 (0.2)	2,298 (0.3)
Cellulitis and abscess of finger and toe (681.XX)	20,060 (3.0)	20,868 (3.0)	22,731 (3.0)	25,174 (3.1)	27,833 (3.2)
Impetigo (684.XX)	1,221 (0.2)	1,084 (0.2)	1,250 (0.2)	1,337 (0.2)	1,481 (0.2)
Infection (chronic) of amputation stump (997.62)	13,431 (2.0)	14,874 (2.1)	15,100 (2.0)	14,459 (1.8)	16,014 (1.8)
Other cellulitis and abscess (682.XX)	346,270 (51.3)	358,884 (51.2)	392,422 (51.8)	428,274 (52.8)	464,016 (53.4)
Other local infections of skin (686.XX)	3,605 (0.5)	3,302 (0.5)	3,419 (0.5)	3,755 (0.5)	4,167 (0.5)
Deeper and/or healthcare-associated infections	274,549 (40.7)	287,736 (41.0)	307,300 (40.6)	320,580 (39.5)	339,337 (39.0)
Abscess of anal and rectal region (566)	20,511 (3.0)	20,273 (2.9)	22,772 (3.0)	23,892 (3.0)	24,655 (2.8)
Chronic ulcer of other specified sites (707.8)	132 (0.0)	75 (0.0)	73 (0.0)	68 (0.0)	62 (0.0)
Chronic ulcer of unspecified site (707.9)	2,136 (0.3)	2,191 (0.3)	2,389 (0.3)	2,666 (0.3)	2,465 (0.3)
Decubitis ulcer (707.0)	37,116 (5.5)	38,637 (5.5)	36,816 (4.9)	38,167 (4.7)	39,706 (4.6)
Infection due to vascular device (996.62)	64,262 (9.5)	73,051 (10.4)	78,289 (10.3)	81,970 (10.1)	91,804 (10.6)
Pilonidal cyst with abscess (685.0)	2,262 (0.3)	2,167 (0.3)	2,447 (0.3)	2,496 (0.3)	2,604 (0.3)
Postoperative wound infection (998.5X)	115,694 (17.1)	120,344 (17.2)	134,783 (17.8)	138,393 (17.1)	144,768 (16.6)
Posttraumatic wound infection, NEC (958.3)	2,748 (0.4)	2,239 (0.3)	1,664 (0.2)	2,326 (0.3)	2,843 (0.3)
Ulcers of lower limbs, except decubitis (707.1X)	29,688 (4.4)	28,759 (4.1)	28,067 (3.7)	30,602 (3.8)	30,431 (3.5)
Often fatal infections	10,232 (1.5)	9,400 (1.3)	10,057 (1.3)	10,965 (1.4)	10,341 (1.2)
Gangrene (785.4)	5,628 (0.8)	4,814 (0.7)	5,155 (0.7)	5,546 (0.7)	4,331 (0.5)
Necrotizing fasciitis (728.86)	4,604 (0.7)	4,586 (0.7)	4,902 (0.7)	5,419 (0.7)	6,010 (0.7)
Hospital region					
Northeast	142,316 (21.1)	155,948 (22.2)	158,649 (20.9)	174,481 (21.5)	171,399 (19.7)
Midwest	152,132 (22.5)	159,442 (22.7)	173,353 (22.9)	175,144 (21.6)	193,602 (22.3)
South	253,388 (37.5)	269,323 (38.4)	297,122 (39.2)	316,028 (39.0)	351,721 (40.4)
West	127,102 (18.8)	116,958 (16.7)	128,734 (17.0)	145,115 (17.9)	153,056 (17.6)
Hospital location†					
Urban	570,554 (84.5)	592,908 (84.5)	645,261 (85.1)	691,795 (85.3)	755,095 (86.8)
Rural	103,664 (15.4)	108,763 (15.5)	112,597 (14.9)	118,563 (14.6)	114,682 (13.2)

*SSTI, skin and soft tissue infection; ICD-9-CM, International Classification of Diseases, 9th Revision, Clinical Modification; NEC, not elsewhere classified. Numbers are given as no. (%) admissions; N values for each year are provided. Source: Healthcare Cost and Utilization Project National Inpatient Sample, 2000–2004. †Percentages may not add up to 100% due to missing data.

## Conclusions

Until recently, CA-MRSA was relatively uncommon. In 2000, for example, these isolates accounted for only 3% of staphylococcal isolates submitted to Minnesota laboratories (*4*). In recent years, however, CA-MRSA has become a major cause of SSTIs. The prevalence of MRSA among patients with SSTIs who sought treatment at 1 Los Angeles area emergency department increased from 29% in 2001 to 64% in 2004 (*5*).

While the estimated total number of SSTI admissions to US acute-care hospitals increased by ≈29% during 2000–2004, admissions for infectious pneumonia were largely unchanged. We are unaware of any other data to which our findings might be directly compared. Our results appear to be consistent, however, with those of previous studies that have reported an increasing prevalence of illness attributable to *S. aureus* infections in general and to MRSA in particular (*6*,*7*). Although we could not establish in our study whether the increase in the number of SSTI hospital admissions was a result of the growing prevalence of CA-MRSA (the HCUP-NIS does not report microbiologic data), we suspect that the 2 phenomena are closely linked—especially in light of the absence of any similar increase in hospital admissions for pneumonia, the most common community-associated infection requiring hospitalization. We therefore believe that the clinical and economic effects of CA-MRSA SSTIs are substantial and growing, and that this increase should be a focus of additional research.
